# Two-Year Follow-Up of 4-mm-Long Implants Used as Distal Support of Full-Arch FDPs Compared to 10-mm Implants Installed after Sinus Floor Elevation. A Randomized Clinical Trial

**DOI:** 10.3390/ijerph18073846

**Published:** 2021-04-06

**Authors:** Fabio Rossi, Lorenzo Tuci, Lorenzo Ferraioli, Emanuele Ricci, Andreea Suerica, Daniele Botticelli, Gerardo Pellegrino, Pietro Felice

**Affiliations:** 1 Department of Biomedical and Neuromotor Science, University of Bologna, 40126 Bologna, Italy; farossi@libero.it (F.R.); lorenzotuci90@gmail.com (L.T.); lorenzoferraioli@gmail.com (L.F.); manuricci@libero.it (E.R.); pietro.felice@unibo.it (P.F.); 2 ARDEC Academy, 47923 Rimini, Italy; andreea.suerica@gmail.com (A.S.); daniele.botticelli@gmail.com (D.B.)

**Keywords:** short implants, full arch fixed dental prostheses, sinus floor elevation

## Abstract

Background: In edentulous patients, bone resorption cannot allow the installation of standard implants and it is demanded to use short implants in the residual alveolar bone or longer implants in grafted bone. Aim: To compare the survival and bone level changes of standard plus short 4-mm implants used as distal support of a maxillary full-arch fixed dental prostheses (FDPs) with standard (10-mm) implants placed in association with a bilateral sinus floor augmentation procedure. Material and Methods: Full-arch FDPs supported by six implants were randomly placed in both groups. In the control group, all implants were 10 mm long and 4.1 mm in diameter. The distal implant in both sides of the maxilla was installed after 4 months from bilaterally sinus floor elevation. In the test group (short group), the distal implant in both sides of the maxilla was 4 mm long and 4.1 mm in diameter. No sinus floor elevations were performed in the test group. Clinical assessments and X-rays were taken at prosthesis delivering and after 6, 12, 18, and 24 months. Patient-reported outcome measures (PROMs) were also evaluated before surgery and after 6, 12, and 24 months. Results: The changes over time of the bone level for the short implants were −0.01 ± 0.11 mm, −0.04 ± 0.13 mm, −0.17 ± 0.29 mm, and −0.28 ± 0.37 mm after 6, 12, 18, and 24 months from prosthesis delivering, respectively. For the standard implants, bone changes were −0.21 ± 0.33 mm (*p* = 0.103), −0.30 ± 0.32 mm (*p* = 0.023), −0.40 ± 0.37 mm (*p* = 0.144), and −0.54 ± 0.49 mm (*p* = 0.128), respectively. A statistically relevant difference was found only at 12 months after loading between the two groups. Conclusions: Similar results on implant survival rate and marginal bone loss were observed for the short and standard implants, placed in association with a bilateral sinus floor augmentation procedure, used as distal support of a maxillary full-arch FDP. A statistically relevant difference was found only at 12 months after loading between the two groups (*p* = 0.023).

## 1. Introduction

Anatomical limitations to implant insertion in fully edentulous arches are frequently encountered in the posterior regions of the jaws, due to the presence of the mandibular canal and of the maxillary sinus. In such situations, the installation of standard implants (≥10 mm) could be impossible. An alternative might be the use of the so-called “tilted implants” that might allow the use of standard implants [1]. Vertical augmentation [2] procedures or sinus floor elevation [3,4] with a different approach [5] might allow the installation of standard implants as well. Another alternative is the use of short implants, as recommended in a consensus conference [6] for the lower occurrence of complications of this treatment compared to the use of longer dental implants installed in augmented sinus. Another consensus report concluded that the use of ≤6-mm-long implants are a valid option to be used as alternative to augmentation procedures to reduce morbidity incidence [7]. A recent article reported a high survival rate (91.7%) after 10 years of loading of 6-mm-long implants supporting single fixed dental prostheses (FDPs) in the posterior regions of both jaws [8]. Recently, 4-mm-long implants with a standard diameter have been used for the rehabilitation of the posterior edentulous mandible with favorable results [9,10]. However, randomized control trials (RCTs) reporting the results from full-arch FDPs that included 4-mm-long implants placed in the posterior regions of the maxilla have not been published yet. Hence, we compared the survival and bone level changes of 4-mm-implants used as distal support of a maxillary full-arch FDPs with control (10 mm) implants placed in association with a bilateral sinus floor augmentation procedure.

## 2. Materials and Methods

The Declaration of Helsinki was followed, and the protocol was approved by the Ethical Committee Interaziendale Bologna-Imola (protocol #15052; 9 September 2015).

All procedures, timing, and complications were systematically explicated to the included participants, and signed informed consents were collected. The Consort checklist was followed for this report (http://www.consort-statement.org/, accessed on 22 May 2019). The study was registered in the ClinicalTrials.gov with the following identifier: NCT03958448.

### 2.1. Study Design

This research is an RCT where short dental implants 4 mm long are compared with longer dental implants 10 mm long in association with SFE- Sinus Floor Elevation. Patients recruited needed full-arch fixed maxilla rehabilitation. Patients suffering severe atrophy in the distal part of the maxilla and two approaches were established. Test patients received super-short 4-mm-long implants as distal support of the FDPs. On the other side, control patients received the same fixed prosthesis rehabilitation, but in the distal atrophic maxilla before placing 10-mm-long implants as distal support, bilateral SFE was performed. Results during healing time and follow-up were carefully registered. 

### 2.2. Study Population

The present study was a parallel randomized control trial initially programmed to include 20 participants. Two groups were randomly arranged, the control and the short groups. Full-arch FDPs supported by six implants were randomly placed in both groups. In the control group, all implants were 10 mm long and 4.1 mm in diameter. The distal implant in both sides of the maxilla was installed 4 months after bilateral sinus floor elevation. In the test group (short group), the distal implant was 4 mm long and 4.1 mm in diameter in both sides of the maxilla. No sinus floor elevations were performed in the test group.

Patients recruitment and all clinical procedures were performed at the Department of Biomedical and Neuromotor Sciences, Dental Clinic, University of Bologna, BO, Italy.

The following inclusion criteria were adopted:

- Edentulous maxilla.
- Willing to receive a full arch fixed restoration in the maxilla.
- Latest extraction at least 8 weeks before implant insertion.
- Sinus floor height included between 4 to 6 mm.
- Bone width in the distal segments enough to allow the insertion of a 4-mm-long implant of standard diameter.
- In the anterior maxilla (from first premolar to first premolar), bone width enough to allow the insertion of 10-mm-long implants of standard diameter.

Minor horizontal augmentations with GBR- Guided Bone Regeneration procedures were allowed in the anterior maxilla.

Moreover, the opposing arch had to present one of the following conditions:

- Natural dentition (at least 10 elements from 3.5 to 4.5).
- FDPs – Fixed dental prosthesis of at least 10 elements (from 3.5 to 4.5) supported by teeth or implants.
- Implant-supported or teeth-supported overdentures.
- Adequate partial removable prostheses.

Exclusion criteria:

- Presence of conditions requiring prophylactic use of antibiotics (e.g., history of rheumatic heart disease, bacterial endocarditis, cardiac valvular anomalies, prosthetic joint replacements).
- Major systemic diseases, or medical conditions requiring prolonged use of steroids, or alcoholism or chronic drug abuse.
- Current pregnancy or breastfeeding women.
- Smokers >10 cigarettes per day.
- Physical handicaps that would interfere with the ability to perform adequate oral hygiene.
- Immunocompromised patients including patients infected with HIV.
- Conditions or circumstances, in the opinion of the investigator, which would prevent completion of study participation or interfere with analysis of study results, such as history of noncompliance, or unreliability.
- Patients with an ongoing or previous treatment with bisphosphonates (for at least 2 months for oral therapy or 6 months for IV injection).
- Local inflammation, including untreated periodontitis.
- Pre-cancerous oral lesions.
- History of local irradiation therapy.
- Severe bruxism or clenching habits.
- Patients with inadequate oral hygiene or unmotivated for adequate oral home care.
- Previous GBR or GTR Guided Tissue Regeneration treatment at the implant site.
- Total removable prosthesis in the lower arch.

### 2.3. Sample Calculation

In the absence of sound data on bone level changes on short implants at the time of the proposal, the present was structured as a pilot study. A bone change of 1 mm over time was proposed to be clinically relevant. With a standard deviation of 0.5 mm, a power of 0.9, and an α = 0.05, ten patients each group were calculated to be enough to disclose differences, including possible dropouts.

### 2.4. Randomization and Assignment Concealment

A statistician not involved in the study performed the randomization in blocks of four and the assignments were sealed in coded and opaque envelopes that were opened at the time of the enrollment of each patient in the study. The surgeon was blinded about the allocation of the treatment until the time of the installation. The patients were blinded about the treatment received.

### 2.5. Implants and Biomaterial Used

The implants were produced by Institute Straumann AG, Basel, Switzerland.

A natural bovine bone grafting material (Cerabone granules 1–2 mm, Botiss biomaterial GmbH, Zossen, Germany) was used for sinus floor elevation.

A porcine dermis collagen membrane (Collprotect membrane, Botiss biomaterial GmbH, Zossen, Germany) was used to cover the antrostomy.

Bone fillers and collagen membranes are distributed by Institute Straumann AG, Basel, Switzerland.

### 2.6. Surgical Procedures in the Short Group (Test)

**Figure 1:**
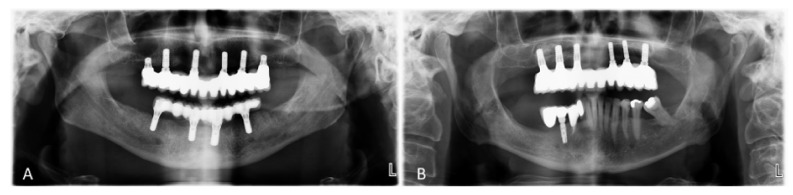
Panoramic X-ray after treatment. Short (**A**) and control implants (**B**) were loaded with fixed full-arch fixed dental prostheses (FDPs). Bilaterally, the most posterior implants were 4 mm long (**A**) or 10 mm long (**B**) that were installed after sinus floor augmentation.

In each side of the posterior region of the maxilla, one 4-mm-long and 4.1 mm in diameter tissue level implant, with a neck of 1.8 mm height, was installed. In the frontal region, four bone level implants, 10 mm long and 4.1 mm or 3.3 mm in diameter, were used (Figure 1A). A non-submerged healing was allowed.

### 2.7. Surgical Procedures in the Standard Group (Control)

Bilateral sinus floor elevations using a lateral access were performed using Cerabone as filler material and Collprotect to cover the antrostomy. After 4 months of healing, one bone level implant, 10 mm long and 4.1 mm in diameter, was installed into each augmented sinus. In the frontal region (included between the second premolars), four bone level implants, 10 mm long and 4.1 mm or 3.3 mm in diameter, were used (Figure 1B). A non-submerged healing was allowed.

### 2.8. Maintenance Care of the Patients

Amoxicillin and clavulanic acid were administrated per os before and for the following 6 days. As painkiller, Ibuprofen 600 mg per os was suggested if needed.

### 2.9. Prosthetic Procedures

During the healing, a temporary removable prosthesis was provided to the patients.

**Figure 2:**
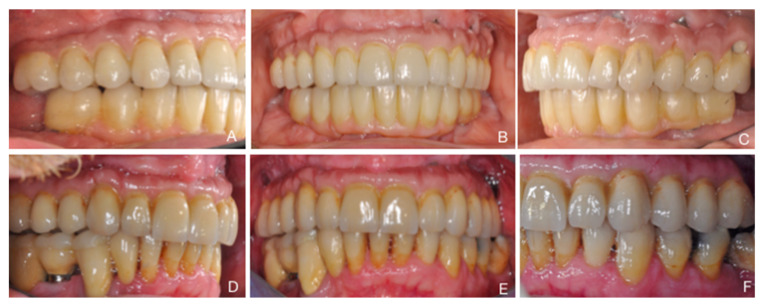
Clinical views, frontals and laterals, of fixed full-arch FDPs. (**A**–**C**) Short implant group. (**D**–**F**) Control implant group, with sinus floor elevation.

Six weeks after implant installation, impressions were taken and a fixed metal ceramic full-arch screw-retained prosthesis was fabricated and delivered (Figure 2A–F).

### 2.10. Clinical Evaluation

Bone quality according to Lekholm and Zarb [11] was subjectively evaluated and the insertion torque was assessed using a ratchet device (Institute Straumann AG, Basel, Switzerland), and categorized as follows: 0 ≤ 15, 15 < x < 35, and ≥35 Ncm. Bleeding on probing [12] and probing depths were assessed around short and control implants at prosthesis delivering and after 6, 12, 18, and 24 months. The highest probing depth was recorded and categorized as follows: 1–3 mm, 4–5 mm, ≥6 mm.

### 2.11. Radiographic Measurements

X-rays applying a parallel technique were taken at implant installation, at prosthesis delivering (baseline) and after 6, 12, 18, and 24 months. The measurements on the digital X-rays were carried out twice by the same operator (L.T.) using the software Planmeca ROMEXIS® (Helsinki, Finland). The calibration of the measurements on the software was obtained using the full length of the implant. The operator presented an intra-class coefficient of correlation for intra-rater agreement >0.93 for marginal bone level radiographic analysis.

The distance between the implant margin and the first bone to implant contact was measured at the mesial and distal aspects of the short and standard implants.

### 2.12. PROMs Evaluation

The PROMs (patient-reported outcome measures) [13] were evaluated using a standardized questionnaire (Oral Health Impact Profile-20E; OHIP-20E) that included 20 questions and to each question the patient had to give a score. The frequency ranged between 1 (always) to 6 (never), and the questions were related to functional limitation, physical pain, physical disability, psychological discomfort, and social disability. The assessments were performed before surgery and after 6, 12, and 24 months. Mean values were calculated per each item and a total score for each group was assessed.

### 2.13. Data Analysis

Only the distal implants were included in the analysis. Mean values were obtained from the double measurements made at the mesial and distal aspects of each implant. Subsequently, mean values were calculated between the distal and mesial measurements for each implant.

At the tissue level implants, the height of the polished neck (1.8 mm) was subtracted to obtain the level of the coronal margin of the rough surface. The marginal bone levels reported in the present article were related to the position of the coronal marginal rough surface. Marginal bone changes were calculated and differences between groups were evaluated at implant level using the U Mann–Whitney test included in the Statistics software Stata (IBM Inc., Chicago, IL, USA). The level of significance was set at α = 0.05.

The primary variable was the change of marginal bone level between the baseline and 1 and 2 years of follow-up.

## 3. Results

**Figure 3:**
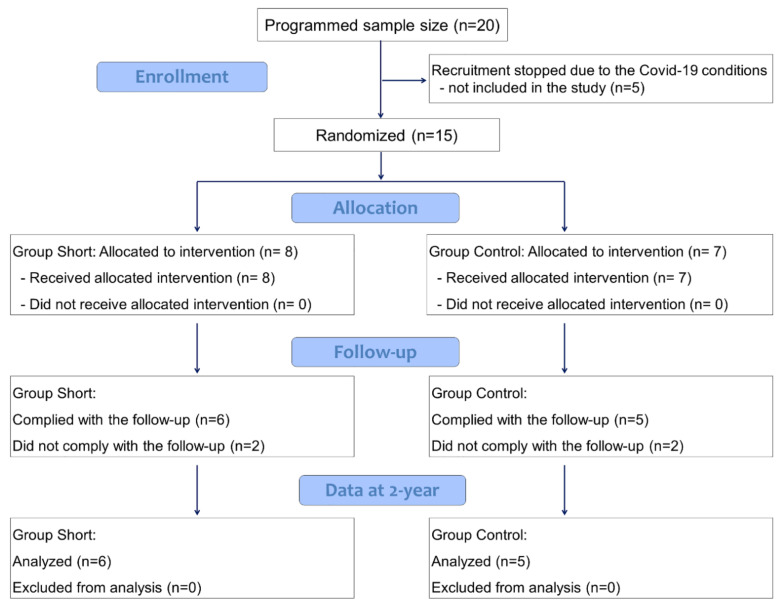
CONSORT 2010 flow diagram.

The study started on 9 September, 2015 and it was interrupted in September 2020. Recruitment stopped due to the COVID-19 conditions, and five patients of the planned 20 were not included in the study. At that date, a total of fifteen patients were included. Considering 15 patients enrolled, only 11 patients, six short and five control, reached the 2-year follow-up, while four participants were excluded from analysis because they did not comply with the requirement in terms of follow-up (Figure 3, Table 1).

No complications were detected during healing. No implant in the distal region of the FDPs was lost, while one implant was lost in the frontal region and replaced in a patient of the control group. In the same patient, another frontal implant presented signs of peri-implantitis after about 18 months of function and was surgically treated. The radiographic documentation was available for evaluation in 12 implants of the short group and 10 implants of the control group.

The clinical assessments showed a maximum probing depth of ≤4 mm in both groups in all periods evaluated. At the baseline, the peri-implant mucosa showed bleeding on probing at one implant site of the short group. After two years of function, 3 out of 12 implant sites of the short group and one out of 10 implant sites of the control group presented bleeding on probing.

No technical complications occurred after 2 years of function.

Radiographic evaluation (Table 2).

**Figure 4:**
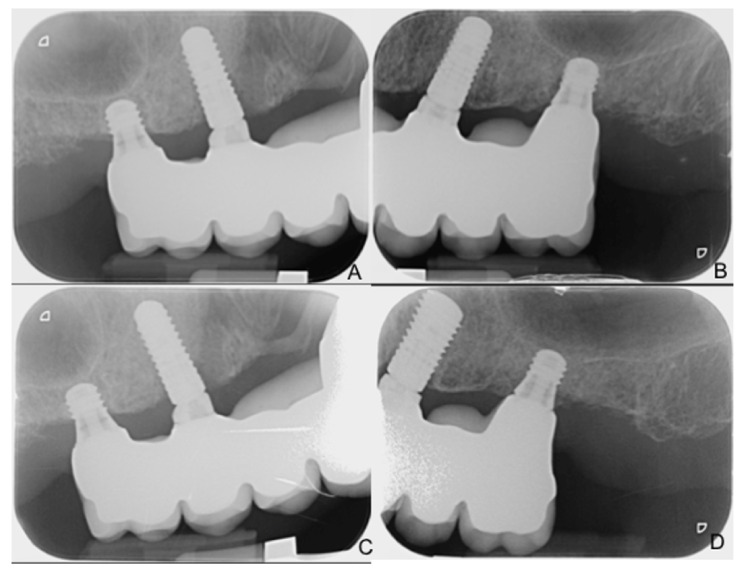
X-rays illustrating the situation of the short implant group immediately after loading (**A**,**B**), and after 24 months of loading (**C**,**D**).

**Figure 5:**
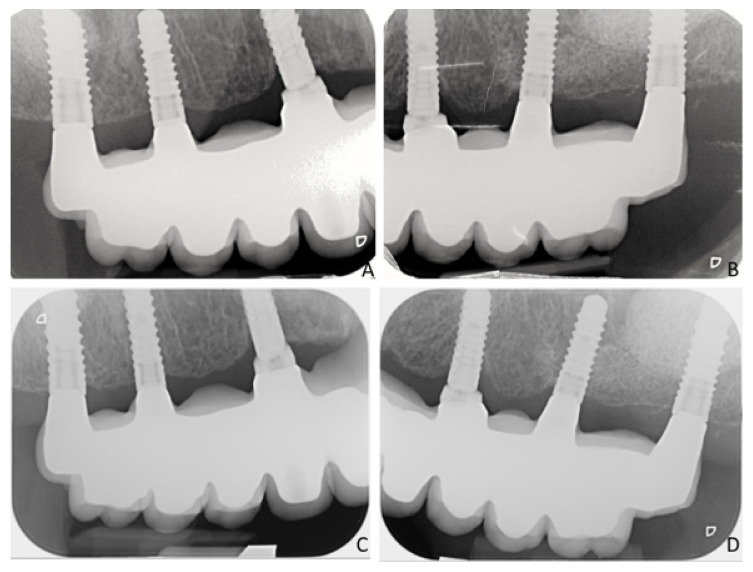
X-rays illustrating the situation of the control implant group immediately after loading (**A**,**B**), and after 24 months of loading (**C**,**D**).

At the baseline (prosthesis delivering), the mean bone level (MBL) was 0.17 ± 0.41 mm with regards to the short implants (Figure 4A,B), while for the control implants was 0.28 ± 0.21 mm (*p* = 0.007) (Figure 5A,B).

The changes over time of the bone level for the short implants were −0.01 ± 0.11 mm, −0.04 ± 0.13 mm, −0.17 ± 0.29 mm, and −0.28 ± 0.37 mm after 6, 12, 18, and 24 months from prosthesis delivering, respectively (Figure 4C,D). For the control implants, bone change levels were −0.21 ± 0.33 mm (*p* = 0.103), −0.30 ± 0.32 mm (*p* = 0.023), −0.40 ± 0.37 mm (*p* = 0.144), and −0.54 ± 0.49 mm (*p* = 0.128), respectively (Figure 5C,D). A statistically relevant difference was found only at 12 months after loading between the two groups (*p* = 0.023); at the very end of the follow-up, no statistical differences were found between the two groups.

### PROMs

The PROMs evaluation revealed a general satisfaction of the patients in both groups about functional limitation, physical pain, physical disability, psychological discomfort, and social disability after the rehabilitation (Table 3A,B).

## 4. Discussion

No implants were lost after 2 years’ follow-up. The survival rate of 4-mm implants used as distal support of maxillary full-arch FDPs was similar to that of 10-mm implants placed in association with a bilateral sinus floor augmentation procedure.

This result agrees with other clinical studies [9,10,14,15,16,17]. In a randomized controlled trial (RCT) [17], eleven patients were rehabilitated bilaterally with FDPs supported by two to four 4-mm-long implants or by 8- or 10-mm-long implants installed in regenerated bone. After one year of follow-up, no implants were lost. In another RCT [15], ten patients with edentulous mandible were rehabilitated with a screw-retained full complete denture. The prosthesis was supported by 10-mm-long implants installed in the intra-foramina region, and two 4-mm-long implants were installed bilaterally, distally to the foramina. One short implant was lost before loading and was substituted. All patients were rehabilitated and, after one year of follow-up, no further loss of implants was registered.

In a multicentric RCT [9], eighty patients with posterior edentulism were included. Forty patients presented 5–6-mm alveolar bone height above the mandibular canal and forty patients presented 4–5-mm of alveolar bone height below the maxillary sinus. The patients were randomly assigned either to receive 4-mm-long implants or an augmentation procedure. After one year of loading, one long implant and two short implants were lost in the mandible, while in the maxilla, three short implants and seven long implants were lost.

In another multicenter RCT [16], one hundred and fifty patients with an edentulous posterior region in the mandible and/or in the maxilla were rehabilitated with screw-retained fixed prostheses supported by implants. A minimum height of 12.5 mm of alveolar bone above the mandibular canal or 11.5 mm below the sinus floor was required. Either short (4-mm) or ≥8.5-mm-long implants were randomly assigned to the patients. After one year of loading, three short and two long implants were lost before loading and replaced. No further loss of implants occurred after loading.

In a prospective study [10], twenty-eight patients were rehabilitated in the posterior mandible with FDPs supported by 4-mm-long tissue level implants. Eighty-six implants were installed, and the patients were followed up to 5 years. Five implants were lost between 3–5 years of loading.

In a systematic review [18], short (≤6 mm) and longer implants (≥10 mm) with sinus floor elevation were compared and analyzed. A total of seven RCTs involving 310 patients were included. The follow-up reached more than three years for several studies. Authors declared that no significant difference with regards to MBL and survival rate were found between each group at each time of the follow-up: 1 up to 3 years and more than 3 years.

In the present study, at baseline, the marginal bone level was located more apically at the control long implants (0.28 mm) compared to the short implants (0.17 mm). The difference was statistically significant (*p* = 0.007). This might be related to an unintentional deeper positioning of the transmucosal short implant compared to the 10-mm-long implant, in an attempt to gain a better primary stability. After one year of loading, 0.04 mm and 0.30 mm of marginal bone loss was found for the short and control implants, respectively (*p* = 0.023). After 2 years of loading, the loss of marginal bone was 0.28 mm and 0.54 mm for the short and control implants, respectively (*p* = 0.128). The encouraging outcomes from the present study support the data reported in the studies mentioned above. In a prospective multicenter study [10], after one year of loading, similar loss of marginal bone was reported for both short and long implants, ranging from 0.30 mm to 0.63 mm, and from 0.47 mm to 0.72 mm, respectively.

These outcomes are also in agreement with other studies that compared 6-mm- with 10-mm-long implants supporting single crowns [19]. Only ~0.1 mm and <0.2 mm of bone loss was observed after 1 and 5 years of loading at both short and long implants. In that study, three short implants were lost after loading. Similar minor loss of marginal bone was observed in both groups. However, a higher loss of implants occurred in the short, compared to the long, groups. The stability of the marginal bone and the sudden loss of stability of the implant/crown unit suggested that the loss of short implants probably owed to a fracture of the supporting alveolar bone. Nevertheless, in a prospective study on single crowns supported by 6-mm-long implants, two implants were lost before loading and only one implant was lost after 7 years of loading. However, a mean loss of 0.8 mm was observed after 10 years of function [8].

Ultimately, our results agree with what was reported by a recent literature review [20] where extra-short (4 mm) dental implants are compared with longer (8 mm) dental implants placed in conditions of severe bone atrophy, analyzing rehabilitations with splinted implants, as in our research project. Authors reported at the end of their review that extra-short implants exhibit satisfactory clinical outcomes concerning implant survival rate and MBL when compared to longer implants, with a low number of biological and prosthetic complications. However, this research also suggests the need to proceed with more in-depth analysis and follow-up.

No technical complications were reported in the present study. This is in agreement with other studies that used the same implant system and had very few technical complications [8,10,15,17].

The patients of both groups reported an improvement of the parameters, as evaluated with OHIP-20E, after prosthesis delivering. However, a trend of higher satisfaction was shown in the short group compared to the control group. This might be related to the higher complexity and extended length of the treatment in the latter compared to the former group. In another clinical trial, single crowns supported by either short implants or standard implants in combination with sinus floor elevation were evaluated [21]. After 5 years of function, no statistically significant differences were found in the PROMs between the two groups. The different scores compared to the present study might be related to the different type of restoration and follow-up.

As limitation of the present study, it should be mentioned the different geometry design of the two groups of implants, that might have influenced both the positioning at the surgery and the following healing. Moreover, both short and long implants were connected to a full arch bridge, and this might have contributed to the stability over time. A longer follow-up is needed to confirm the results. As our main focus and outcome in this article were survival rate and bone loss around implants, we did not statistically evaluate the score changes for each question over time of the PROMs questionnaire. We reported the data in a more descriptive way, and this might be a limitation too.

## 5. Conclusions

The present study reported similar results on implant survival rate and marginal bone loss for 4-mm- and 10-mm-long implants, placed in association with a bilateral sinus floor augmentation procedure, used as distal support of a maxillary full-arch FDP.

Analysing OHIP-20E, a much greater satisfaction was registered in both groups after rehabilitation, but higher scores were obtained in the short group.

## Figures and Tables

**Table 1:** Demographic and clinical data.

	Male	Female	Mean Age	Smokers	Maxilla	Bone Type ^1^ I, II, III, IV	Insertion Torque ^1^ (Ncm)	Antagonist ^1^
	3	3	59.8	4 out of 6	16 and 26	Type I: 0 Type II: 0 Type III: 6 Type IV: 6	<15: 0 15 < x< 35: 6 ≥35: 6	ND: 2 FDP: 2 OD: 0 Mix: 8
STANDARD	3	2	60.8	2 out of 5	16 and 26	Type I: 0 Type II: 0 Type III: 6 Type IV: 4	<15: 0 15 < x < 35: 6 ≥35: 4	ND: 2 FDP: 0 OD: 2 Mix: 6

ND: natural dentition, FDP: fixed dental prosthesis, OD: overdenture, Mix: natural dentition and implants. ^1^: Assessed for each implant.

**Table 2:** Radiographic evaluation. Mean bone level (MBL), distance between the implant margin (M), and the first bone-to-implant contact (B). Evaluation performed at implant level.

	MBL Short	MBL Control	Progressive Changes Short	Progressive Changes Control
Prosthesis delivering (BL)	0.17 ± 0.41	0.28 ± 0.21	NA	NA
6 months	0.18 ± 0.34	0.49 ± 0.44	−0.01 ± 0.11	−0.21 ± 0.33
12 months	0.21 ± 0.35	0.58 ± 0.44	−0.04 ± 0.13	−0.30 ± 0.32
18 months	0.34 ± 0.35	0.68 ± 0.51	−0.17 ± 0.29	−0.40 ± 0.37
24 months	0.44 ± 0.37	0.84 ± 0.68	−0.28 ± 0.37	−0.54 ± 0.49

**Table 3:** (**A**) Control group. Mean values (score) and SDs of Oral Health Impact Profile (OHIP) domains for maxillary full-arch FDPs in control group at pre-surgical time, 6 months, and 12 months. (**B**) Short group. Mean values (score) and SDs of OHIP domains for maxillary full-arch FDPs in short group at pre-surgical time, 6 months, and 12 months.

(**A**)					
	**Functional Limitation**	**Physical Pain**	**Physical Disability**	**Psychological Discomfort**	**Social Disability**
Mean Pre-Sur SD Pre-Sur	3.2 0.6	4.5 0.1	3.1 0.7	3.6 0.5	4.1 1.0
Mean 6 m SD 6 m	4.9 0.2	5.0 0.8	5.3 0.4	5.4 0.3	5.8 0.2
Mean 12 m SD 12 m	4.9 0.1	5.2 0.6	5.6 0.4	5.5 0.2	5.8 0.2
Mean 24 m SD 24 m	5.5 0.6	5.2 0.3	5.7 0.3	5.8 0.2	4.9 2.4
(**B**)					
	**Functional Limitation**	**Physical Pain**	**Physical Disability**	**Psychological Discomfort**	**Social Disability**
Mean Pre-Sur SD Pre-Sur	3.3 0.7	4.9 0.0	3.6 1.0	3.9 0.7	4.9 0.6
Mean 6 m SD 6 m	6.0 0.1	5.8 0.1	5.9 0.1	5.8 0.1	5.9 0.1
Mean 12 m SD 12 m	5.9 0.2	5.9 0.1	6.0 0.0	6.0 0.0	6.0 0.0
Mean 24m SD 24m	5.9 0.2	5.8 0.1	6.0 0.0	6.0 0.0	6.0 0.0

Pre-Sur = Pre-surgical time (n = 5); 6 m = 6 months (n = 5); 12 m = 12 months (n = 5); 24 m = 24 months (n = 5). Higher values correspond to higher patient satisfaction (A). Pre-Sur = Pre-surgical time (n = 6); 6 m = 6 months (n = 6); 12 m = 12 months (n = 6); 24 m = 24 months (n = 6). Higher values correspond to higher patient satisfaction (B).

## Data Availability

The study was registered in the ClinicalTrials.gov with the following identifier: NCT03958448.
